# Effectiveness of Mindfulness-Based Cognitive Therapy With Follow-Up Sessions for Pharmacotherapy-Refractory Anxiety Disorders: Protocol for a Feasibility Randomized Controlled Trial

**DOI:** 10.2196/33776

**Published:** 2022-01-21

**Authors:** Mitsuhiro Sado, Akira Ninomiya, Maki Nagaoka, Akihiro Koreki, Naho Goto, Yohei Sasaki, Chie Takamori, Teppei Kosugi, Masashi Yamada, Sunre Park, Yasunori Sato, Daisuke Fujisawa, Atsuo Nakagawa, Masaru Mimura

**Affiliations:** 1 Department of Neuropsychiatry Keio University School of Medicine Tokyo Japan; 2 Center for Stress Research Keio University Tokyo Japan; 3 National Hospital Organization Shimofusa Psychiatric Medical Center Chiba Japan; 4 Faculty of Nursing and Medicine Care Keio University Tokyo Japan; 5 Clinical and Translational Research Center Keio University Hospital Tokyo Japan; 6 Department of Preventive Medicine and Public Health Keio University School of Medicine Tokyo Japan; 7 Palliative Care Center Keio University Hospital Tokyo Japan

**Keywords:** mindfulness-based cognitive therapy, anxiety disorders, long-term effects, randomized controlled trial, cost-effectiveness

## Abstract

**Background:**

Augmented mindfulness-based cognitive therapy (MBCT) with treatment as usual (mainly pharmacotherapy) is reported to be effective after treatment for anxiety disorders. However, whether its effectiveness persists in the long term is unclear.

**Objective:**

This study aims to examine the feasibility, acceptability, and effectiveness of a follow-up program by conducting a feasibility randomized controlled trial (RCT) that compares augmented MBCT with follow-up sessions and that without follow-up sessions in preparation for a definitive RCT.

**Methods:**

The study involves an 8-week MBCT with a 10-month follow-up. Patients aged 20 to 65 years who meet the Diagnostic and Statistical Manual of Mental Disorders, 4th edition (DSM-IV) criteria for panic disorder, agoraphobia, or social anxiety disorder, which is not remitted with usual treatment for at least 4 weeks, will be included in the study and randomly allocated to receive augmented MBCT with follow-up sessions or augmented MBCT without follow-up sessions. For this feasibility RCT, the primary outcomes are (1) study inclusion rate, (2) dropout rate, (3) attendance rate, and (4) mean and standard deviation of several clinical measures at 8 weeks and 5, 8, and 12 months.

**Results:**

We started recruiting participants in January 2020, and 43 participants have been enrolled up to January 2021. The study is ongoing, and data collection will be completed by May 2022.

**Conclusions:**

This study is novel in terms of its design, which compares augmented MBCT with and without follow-up sessions. The limitations of the trial are as follows: (1) mixed participants in terms of the delivery mode of the intervention, and (2) lack of a pharmacotherapy-alone arm. Owing to its novelty and significance, this study will provide fruitful knowledge for future definitive RCTs.

**Trial Registration:**

UMIN Clinical Trials Registry UMIN000038626; https://tinyurl.com/2p9dtxzh

**International Registered Report Identifier (IRRID):**

DERR1-10.2196/33776

## Introduction

### Background

Anxiety disorders are the most prevalent mental disorders worldwide [1]. The global 12-month prevalence rate is estimated to be 7.3% [2,3], although prevalence varies across regions (eg, from 3.3% to 18.1% [4-6]). The early age of onset [7] and high probability of relapse [8] prolong the course of the disease, negatively affecting patients socially and chronically [9]. The cumulative remission rates are as low as 35% for social anxiety disorders, 42% for panic disorders with agoraphobia, and 50% for generalized anxiety disorders over a 10-year period [10]. Such features of the disorder place a considerable burden on society. In 2015, anxiety disorders were the sixth leading contributor to nonfatal health loss globally, generating a global total of 24.6 million years lost due to disability [11]. The impact becomes more obvious when the burden is converted into a monetary measure. The societal costs of anxiety disorders were 42.3 billion USD in the United States (1990) [12], 8.9 billion GBP (11.9 billion USD) in the United Kingdom (2007) [13], and 2.4 trillion JPY (27 billion USD) in Japan (2008) [14].

Major clinical guidelines suggest pharmacotherapy and cognitive behavioral therapy (CBT) as the recommended treatments for anxiety disorders [15-17]. However, because of an overwhelming shortage of CBT therapists, a limited number of patients (4.5% in the United States) are able to access CBT [18]. Consequently, pharmacotherapy is currently the dominant treatment strategy. Although the effectiveness of pharmacotherapy for anxiety disorders has been confirmed, remission rates remain between 25% and 35% [19]. Therefore, developing a subsequent treatment for pharmacotherapy-refractory patients, which is effectual and cost-effective in the long term, is important.

Mindfulness-based cognitive therapy (MBCT) [20], which integrates mindfulness-based stress reduction (MBSR) programs with the essence of CBT [21], is a candidate option. MBCT cultivates mindfulness and nonjudgmental present-moment awareness, which allows people to become aware of their bodily sensations, feelings, and thoughts. MBCT is normally offered in a group format and could be more efficient than individual CBT.

### What We Already Know

MBCT has a significant favorable effect on anxiety disorders [22-28]. Even in a setting where the majority of patients manifest pharmacotherapy resistance, MBCT augmented with pharmacotherapy appears to be more effective than pharmacotherapy alone at posttreatment [29]. However, its long-term effectiveness is unclear. For treatment-resistant depression, Eisendrath et al [30] showed that the effects of augmented MBCT on the reduction of Hamilton Depression Rating Scale scores at posttreatment disappeared 1 year later. One possible explanation is that termination of the treatment discourages patients from continuing to practice mindfulness meditation posttreatment.

### Rationale for the Study

As Segal et al indicated [31], although the practice time does not directly affect the clinical outcome, it could affect the outcome mediated by the “decentering” skill improved by the meditation practice. Given that the practice time diminishes as the intervention terminates [32], adding follow-up sessions posttreatment would encourage patients to practice meditation continuously, possibly leading to a better outcome through the improvement of the core skill of decentering. Therefore, in anticipation of future definitive randomized controlled trials (RCTs), we decided to conduct a feasibility RCT to compare augmented MBCT (ie, MBCT plus pharmacotherapy) with follow-up sessions and augmented MBCT without follow-up in order to (1) assess the feasibility, safety, and effectiveness of augmented MBCT with follow-up sessions and (2) compare clinical outcomes between the 2 arms.

### Aim

This study aims to examine the feasibility, acceptability, and effectiveness of a follow-up program in an augmented MBCT scheme by conducting a feasibility RCT between augmented MBCT with and that without follow-up sessions.

## Methods

### Participants

The study is being conducted at Keio University Hospital in Tokyo, Japan. We will recruit participants from the Department of Neuropsychiatry. Patients are eligible for the study if they are between the ages of 20 and 65 years; meet the Diagnostic and Statistical Manual of Mental Disorders, 4th edition (DSM-IV) criteria for panic disorder, agoraphobia, or social anxiety disorder, which is not remitted with usual treatment (pharmacotherapy) for at least 4 weeks; and are capable of providing written consent. The exclusion criteria are substance abuse or dependence, antisocial personality disorder, severe suicidality, self-harm, organic brain damage, severe physical illness, and other appropriate factors deemed by the principal investigator. Patients who are unlikely to attend for 12 months (eg, expected to be moving) will be excluded.

### Enrollment

During usual consultation, the psychiatrist will provide brief information on the study with a leaflet and ask the patients about their willingness to participate in the study. If the patients show interest, the study psychiatrist will arrange an appointment for an interview. The study psychiatrist will explain exhaustively the details of the expected benefits and risks of participation in the study, as well as discuss any questions from the candidate participants. The patients will be evaluated for study eligibility by the study psychiatrist or psychologist.

The study psychiatrist or psychologist will assess the diagnosis of the participants using the Japanese version of the Structured Clinical Interview for DSM-IV (SCID) Axis I Disorders [33] under the supervision of MS, who has completed training in the administration of semistructured interviews. Written informed consent will be obtained from eligible participants after the study procedures are explained in detail.

### Baseline Assessment

Participants will be asked to fill a battery of questionnaires relevant to demographic and psychosocial data. Psychological scales include the State-Trait Anxiety Inventory (STAI), Panic and Agoraphobia Scale (PAS), Liebowitz Social Anxiety Scale (LSAS), Experiences Questionnaire (EQ), Short-Form 36-Item Health Survey (SF-36), Scale of Positive and Negative Experience (SPANE), Rosenberg Self-Esteem Scale (RSES), Five Facet Mindfulness Questionnaire (FFMQ), Connor Davidson Resilience Scale (CDRISC), Self-Compassion Scale (SCS), 16-item Quick Inventory of Depressive Symptomatology (QIDS), Generalized Anxiety Disorder Assessment-7 (GAD7), Perceived Stress Scale (PSS), World Health Organization Health and Work Performance Questionnaire (WHO-HPQ), Satisfaction With Life Scale (SWLS), Flourishing Scale (FS), Multidimensional Assessment of Interoceptive Awareness (MAIA), EuroQoL-5 Dimensions (EQ-5D), health care service use (including medication), Hamilton Anxiety Scale (HAM-A), and interoception. All assessments, except for the latter 2 (ie, HAM-A and interoception), are intended to be conducted in a self-report format.

### Randomization

**Figure 1:**
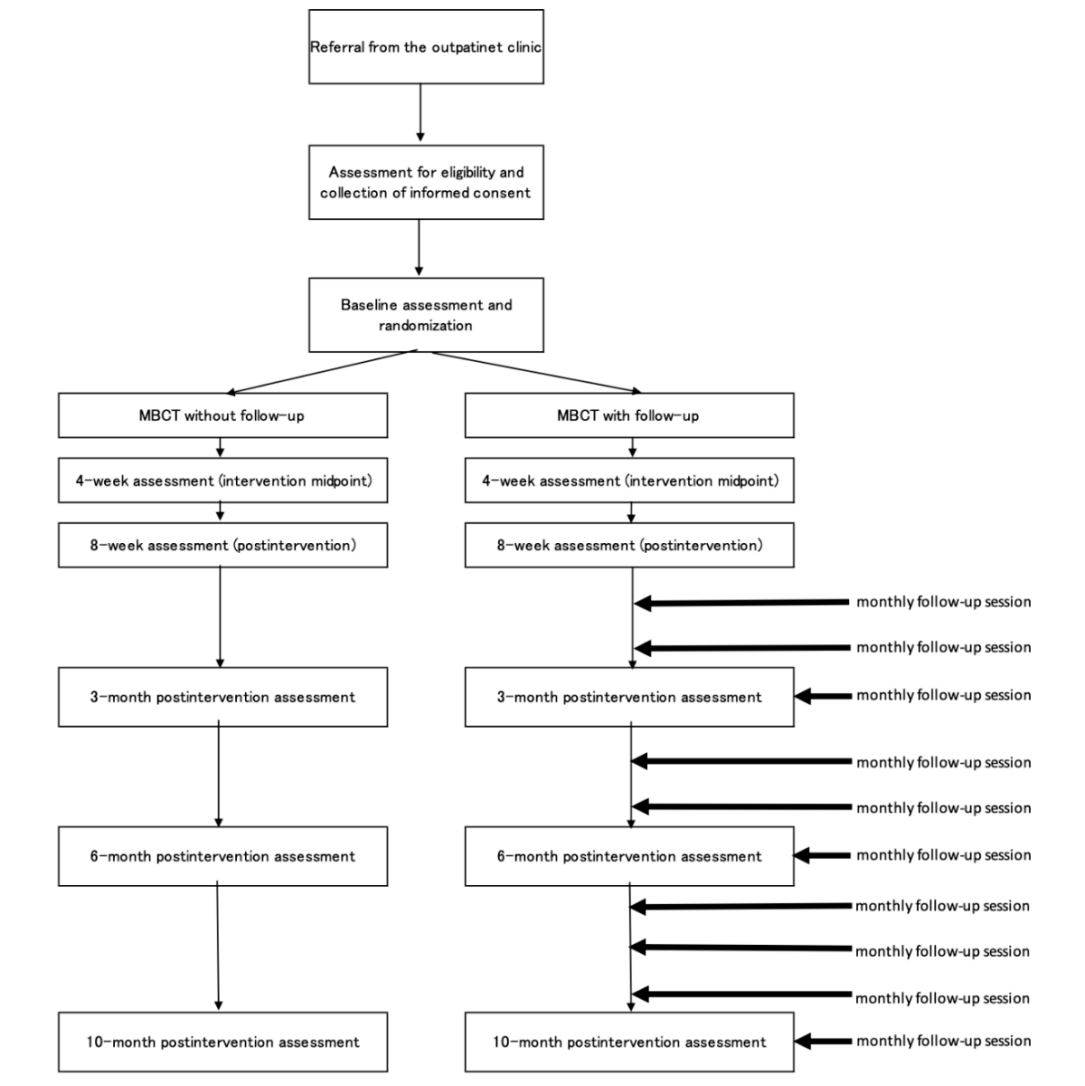
Flow of the study. MBCT: mindfulness-based cognitive therapy.

Eligible participants will be randomly allocated to either the augmented MBCT with follow-up sessions group or MBCT without follow-up sessions group (1:1 ratio). A computer-generated random number stratified by diagnosis (ie, panic disorder/agoraphobia and social anxiety disorder) and baseline score for the STAI will be assigned to each participant. The Project Management Office at Keio Center of Clinical Research, which is an independent institution from the study group, will manage the randomization process. The flow of the recruitment of participants is shown in Figure 1.

### Blinding

The randomization status will not be blinded to both participants and therapists because of the nature of the psychological intervention. The raters blind to the allocation status will perform assessments for interoception and HAM-A. Both participants and therapists will be strongly indicated not to report their treatment allocation at rater-administered assessments. The assessors are independent and not involved in the treatment administration.

### Interventions

#### MBCT With Follow-up Sessions Group

The patients in the intervention group will be offered an 8-week MBCT followed by a 10-month follow-up program. The MBCT consists of 8 weekly sessions in a group format. Each session lasts for 2 hours. In the program, participants practice mindfulness meditation as well as cognitive exercise. Minimum modifications have been made to the original version of MBCT [20] because the study targets patients with anxiety disorders rather than those with depression. Specifically, we have replaced psychoeducation of depression with that of anxiety. Table 1 describes the themes and contents of each session. The participants will be requested to practice mindfulness meditation for approximately 30 minutes daily and to record the duration of time they meditated and the meditation type.

**Table 1:** Themes and content of the program.

Session	Theme	Content
1	Automatic pilot	Psychoeducation: What is mindfulness? Exercise: Mindfulness eating (“raisin exercise”)/body scan Homework: Mindfulness of a routine activity/body scan
2	Dealing with barriers	Psychoeducation: Association of mood and thoughts Exercise: Thoughts and feelings exercise/body scan/mindful breathing meditation Homework: Body scan/breathing meditation/pleasant events calendar
3	Mindfulness of the breath	Psychoeducation: Awareness of mind wandering and focusing on the breath Exercise: Breathing meditation/gentle yoga/mindful walking Homework: Breathing meditation/gentle yoga/mindful walking/unpleasant events calendar
4	Staying present	Psychoeducation: Staying present/about anxiety symptoms^a^ Exercise: Meditation of sounds and thoughts/breathing meditation Homework: Meditation of sounds and thoughts/breathing meditation/3-minute breathing space
5	Allowing/letting be	Psychoeducation: Exploring difficulty Exercise: Breathing meditation/meditation of sounds and thoughts/exploring difficulty Homework: Breathing meditation/meditation of sounds and thoughts/exploring difficulty/3-minute breathing space
6	Thoughts are not facts	Psychoeducation: Cognitive biases Exercise: Breathing meditation/meditation of sounds and thoughts/exploring difficulty Homework: Breathing meditation/meditation of sounds and thoughts/exploring difficulty/3-minute breathing space
7	How can I best take care of myself?	Psychoeducation: Choosing functional behaviors/behavioral activation/identifying triggers Exercise: Mindfulness meditation of sounds and thoughts/breathing meditation Homework: Meditation of sounds and thoughts/breathing meditation/3-minute breathing space plus action plan
8	Using what has been learned to deal with future mood	Personal reflections of course/plans for future practice and strategies for maintaining momentum/farewell Exercise: Body scan/breathing meditation

^a^The lecture relevant to depression has been replaced by that about anxiety in session 4.

After the completion of the 8-week MBCT, the participants in the intervention group will be offered to continue the 10-month follow-up program. The program is designed to consist of 2 elements. The first is the 10 monthly follow-up sessions. The participants will be encouraged to attend 1.5-hour–long monthly follow-up sessions, where they will meditate together and share their experiences of mindfulness in daily life. The second element is the use of mindfulness apps in daily life. Participants in the group will be provided with mindfulness apps developed by the research team to be used during the follow-up period. The participants can access the apps either from a smartphone or PC and stream/download the meditation instructions easily. In addition, they are encouraged to send their mindfulness experiences in daily life to the research team every month. The research team will post and share them with other participants on the apps. The research team will also post relevant articles to support participants in continuing the practice. No regular homework will be offered during the follow-up period. Participants will be encouraged to meditate depending on their needs.

The first (MS), second (A Ninomiya), and third (MN) authors led the sessions. The first author is a qualified MBSR teacher at the University of Massachusetts, with 10 years of experience in mindfulness practice. The other 2 authors have been practicing mindfulness for more than 5 years and have experience in offering MBCT 5 times under the supervision of the first author.

#### MBCT Without Follow-Up Sessions Group

Participants in the control group will also be offered the 8-week MBCT. During the follow-up period, they will be encouraged to continue practicing by themselves. However, no additional intervention is intended to be provided after the 8-week MBCT.

#### Response During the COVID-19 Pandemic

MBCT and follow-up sessions were initially planned to be offered in person. However, to ensure participant safety during the COVID-19 pandemic, classes will be offered online.

### Outcomes

#### Primary Outcomes

In this feasibility RCT, the primary outcomes are the (1) study inclusion rate, (2) dropout rate, (3) attendance rate, and (4) mean and standard deviation of the below clinical measures at 8 weeks and 5, 8, and 12 months.

#### Clinical Outcomes

The primary clinical outcome is the mean and standard deviation of the STAI score in both groups at 8 weeks and 5, 8, and 12 months after the start of the intervention. The mean difference and standard deviation of the STAI score between the groups is also assessed.

The secondary clinical outcomes are PAS, LSAS, EQ, SF-36, SPANE, RSES, FFMQ, CDRISC, SCS, QIDS, GAD7, PSS, WHO-HPQ, SWLS, FS, MAIA, EQ-5D, HAM-A, and interoception scores (baseline, 8 weeks, and 12 months only for HAM-A and interoception); health service use; engagement in meditation practice; and satisfaction with and expectation of the classes.

#### Cost-effectiveness

Cost-effectiveness is assessed by the incremental cost-effectiveness ratio, which represents the incremental cost divided by the incremental effectiveness between the groups. Incremental effectiveness is evaluated using quality-adjusted life years calculated from the results of EQ-5D. The analyses are conducted from a health care system perspective.

### Instruments

#### STAI

The STAI is a commonly used measure of state and trait anxiety. It can be used in clinical settings to diagnose anxiety and distinguish it from depressive syndromes. It has 20 items for assessing trait anxiety and 20 for assessing state anxiety. Possible scores range from 20 to 80. Higher scores indicate higher anxiety [34].

#### PAS

The PAS is a measure of illness severity in patients with panic disorder (with or without agoraphobia). It has 13 items with a 5-point scale, which covers the following 5 subscales: panic attacks, agoraphobic avoidance, anticipatory anxiety, disability, and functional avoidance (health concerns). Higher scores indicate more severity [35].

#### LSAS

This instrument is used to assess patients’ fear in a range of social interactions and performance situations. The scale consists of 24 items, which are categorized into the following 2 elements: performance anxiety (13 items) and social situations (11 items). Scores are between 0 and 144, with higher scores indicating higher social anxiety [36].

#### EQ

The EQ is a 20-item self-report measure using a 5-point Likert scale ranging from 1 (*never*) to 5 (*always*). The total score is between 20 and 100. The scale focuses on decentering, defined as the ability to view the self as separate and different from own thoughts, the capacity for not reacting to negative experiences, and the ability to be self-compassionate. The EQ has been found to be reliable, and convergent and discriminant validities are established for both general and clinical samples. The EQ is also internally consistent, with temporal stability over a 1-month period and good convergent validity [37,38].

#### SF-36

The SF-36 is a 36-item multipurpose health survey to evaluate 8 health domains of functional health and the level of well-being, as well as physical and mental health summary measures and a health utility index. Possible scores for each domain range from 0 to 100, with higher scores indicating a better health status [39].

#### SPANE

This measure is a 12-item scale to assess positive experiences (6 items) and negative experiences (6 items). Because of the generality of items included in the scale, it can not only assess pleasant and unpleasant emotional feelings that are the focus of most scales, but also reflect other conditions, such as interest, flow, positive engagement, and physical pleasure. The positive (SPANE-P) and negative (SPANE-N) scale scores range between 6 and 30. Higher scores indicate a higher positive or negative affective status. The score obtained on subtracting the negative score from the positive score is called the SPANE-B score, which is between −24 and 24 [40].

#### RSES

This is a brief self-rated assessment tool to evaluate self-esteem, self-worth, acceptability, and confidence. It is the most recognized and widely used measure for these metrics. It has 10 items with a Likert scale (1 = *strongly disagree*, 4 = *strongly agree*). The total score ranges from 10 to 40, with higher scores indicating better self-esteem [41].

#### FFMQ

This tool is a self-report questionnaire that assesses mindfulness. It includes 5 factors, which are extracted on the basis of a factor analysis of 5 mindfulness questionnaires developed independently. The 5 facets are observing, describing, acting with awareness, not judging one’s inner experience, and not reacting to one’s inner experience. The total score ranges from 39 to 195, with higher scores representing a better mindfulness status [42].

#### CDRISC

The CDRISC is a brief self-rated assessment to measure resilience. The scale contains 25 items, all of which feature a 5-point Likert scale (4 = *true nearly all of the time*, 0 = *not true at all*). The scale is rated based on how the subjects felt over the past month. The total score ranges from 0 to 100, with higher scores reflecting greater resilience [43].

#### SCS

This scale assesses a person’s ability to be kind and understanding toward themself, as opposed to harsh and self-critical in instances of pain or failure. It includes 29 items and produces scores on 5 subscales (self-kindness, self-judgment, common humanity, isolation, mindfulness, and overidentification). The subscale scores represent the mean of each subscale’s item scores. Participants are asked to answer how often they had certain thoughts and feelings (1 = *rarely* to 5 = *very often or always*). Therefore, each subscale score is between 1 and 5. Higher scores indicate more self-compassion [44].

#### QIDS

The QIDS is a self-rated questionnaire to assess depressive symptoms, which is widely used. The responses to 16 separate items on the QIDS are converted into 9 DSM-IV symptom criterion domains. The total score is between 0 and 27. Higher scores indicate higher levels of depressive symptoms [45].

#### GAD7

GAD7 was developed to ask patients how often they experienced a set of symptoms in the past 2 weeks. Respondents respond using 4 response options on a Likert scale (0 = *not at all* to 3 = *nearly every day*). In addition, an item assessing the duration of anxiety symptoms is included. Therefore, GAD7 scores are between 0 and 21. Scores of 5, 10, and 15 represent mild, moderate, and severe anxiety symptoms, respectively [46].

#### PSS

The PSS was designed to measure the degree to which situations in one’s life are appraised as stressful. The scale has the following 2 versions: the 14-item version (PSS-14) and the 10-item version (PSS-10), with 4 items removed from the 14-item version. We use the PSS-10 in this study. This scale assesses perceived stressful experiences or stress responses in the previous month. Each item is rated on a 5-point Likert scale (4 = *never*, 0 = *very often*) to identify positive experiences or responses. The total score ranges from 0 to 40, with higher scores representing higher stress levels [47].

#### WHO-HPQ

The WHO-HPQ is a self-report instrument designed to estimate the workplace costs of health problems in terms of self-reported sickness absence and reduced job performance (presenteeism). Presenteeism is measured using the following two questions: “On a scale from 0 to 10, where 0 is the worst job performance anyone could have at your job and 10 is the performance of a top worker, how would you rate the usual performance of most workers in a job similar to yours?” and “Using the same 0-10 scale, how would you rate your overall job performance on the days you worked during the past four weeks?” A low presenteeism score indicates poor performance [48].

#### SWLS

This scale is a 5-item self-reported questionnaire to evaluate the cognitive aspect of subjective well-being. Scores for each subscale range from 1 (*strongly disagree*) to 7 (*strongly agree*). The total score ranges from 5 to 35, with higher scores indicating higher satisfaction [49].

#### FS

This scale includes 8 items relevant to significant aspects of human functioning, ranging from positive relationships to feelings of competence, meaning, and purpose in life. Each item is answered on a 7-point scale that ranges from 1 (*strong disagreement*) to 7 (*strong agreement*). Possible scores range between 8 (*strong disagreement* with all items) and 56 (*strong agreement* with all items). Higher scores indicate that respondents viewed themselves positively in important areas of functioning [40].

#### MAIA

The MAIA is a self-report scale for experimental interoception research and for the assessment of mind-body therapies [50]. It is a 32-item self-report instrument to assess interoceptive awareness on the following 8 subscales: noticing, not distracting, not worrying, attention regulation, emotional awareness, self-regulation, body listening, and trusting. Each subscale has 3 to 7 items, each assessed on a 6-point Likert scale (0 = *never*, 5 = *always*). Scores for each subscale range from 0 to 5. Higher scores indicate better interoceptive awareness [51].

#### EQ-5D

EQ-5D is a standardized measure for assessing health-related quality of life. Applicable to a wide range of health conditions and treatments, it provides a simple descriptive profile and a single index value for health status. The score ranges between 0 (death) and 1 (perfect health) [52].

#### HAM-A

HAM-A is a rating scale used to measure the severity of anxiety symptoms. It includes 14 items that measure both psychiatric and somatic anxiety. Each item is scored from 0 (*not present*) to 4 (*severe*), with a total score between 0 and 56. HAM-A has a structured interview guide (Structured Interview Guide for Hamilton Anxiety Scale: SIGH-A) [53,54].

#### Interoception

To assess interoception objectively, we use a heartbeat detection task that has been used and validated worldwide. The participants are asked to wear a pulse oximeter on their finger, which is connected to a PC to evaluate their actual pulse. They are also asked to count the heartbeat felt during various measurement periods. Interoceptive accuracy is measured based on the discrepancy between the number of actual and reported heartbeats [55,56]. The validity and reliability of all measures of the original and Japanese versions, except the Japanese version of the PAS, have been confirmed [34-54,57-74]. With respect to the Japanese version of the PAS, although it shows sufficient reliability, the authors recommend using it as a secondary outcome, because the criterion-related validity indicates “relatively strong correlation” (ie, the correlation coefficient ranges between 0.48 and 0.68). We judge it to be sufficient for use as a secondary outcome.

### Schedule for Assessments

All participants will be requested to fill the self-report measures at 4 weeks (the intervention midpoint) and 8 weeks (postintervention), and at 3, 6, and 10 months postintervention, as well as complete the baseline assessments. HAM-A and interoception will be assessed at baseline (0 weeks), at the end of the MBCT (8 weeks), and at 10 months postintervention. We will allow for a range of ±2 weeks from the scheduled evaluation date for the evaluation during the intervention period and ±4 weeks from the scheduled evaluation date for the evaluation during the follow-up period. For those who are unable to come to the hospital to complete the self-rated scales, we will contact them and ask them to fill out and return the above evaluation items by mail or telephone. The assessment schedule is presented in Table 2.

**Table 2:** Schedule of assessments

	Study period																			
	Screening period	Intervention period									Follow-up period									
Timepoint		1 wk	2 wk	3 wk	4 wk	5 wk	6 wk	7 wk	8 wk	1 mo		2 mo	3 mo	4 mo	5 mo	6 mo	7 mo	8 mo	9 mo	10 mo
Screening (SCID^a^, etc)	✓																			
Informed consent	✓																			
MBCT with f/u^b^		✓	✓	✓	✓	✓	✓	✓	✓	✓		✓	✓	✓	✓	✓	✓	✓	✓	✓
MBCT without f/u^c^		✓	✓	✓	✓	✓	✓	✓	✓											
STAI^d^	✓				✓				✓				✓			✓				✓
PAS^e^	✓				✓				✓				✓			✓				✓
LSAS^f^	✓				✓				✓				✓			✓				✓
EQ^g^	✓				✓				✓				✓			✓				✓
SF-36^h^	✓				✓				✓				✓			✓				✓
SPANE^i^	✓				✓				✓				✓			✓				✓
RSES^j^	✓				✓				✓				✓			✓				✓
FFMQ^k^	✓				✓				✓				✓			✓				✓
CDRISC^l^	✓				✓				✓				✓			✓				✓
SCS^m^	✓				✓				✓				✓			✓				✓
QIDS^n^	✓				✓				✓				✓			✓				✓
GAD7^o^	✓				✓				✓				✓			✓				✓
PSS^p^	✓				✓				✓				✓			✓				✓
WHO-HPQ^q^	✓				✓				✓				✓			✓				✓
SWLS^r^	✓				✓				✓				✓			✓				✓
FS^s^	✓				✓				✓				✓			✓				✓
MAIA^t^	✓				✓				✓				✓			✓				✓
EQ-5D^u^	✓				✓				✓				✓			✓				✓
HAM-A^v^	✓								✓											✓
Interoception	✓								✓											✓
Health service use	✓				✓				✓				✓			✓				✓
Homework engagement		✓	✓	✓	✓	✓	✓	✓	✓	✓		✓	✓	✓	✓	✓	✓	✓	✓	✓

^a^SCID: Structured Clinical Interview for Diagnostic and Statistical Manual of Mental Disorders, 4th edition disorders.

^b^MBCT with f/u: mindfulness-based cognitive therapy with follow-up sessions.

^c^MBCT without f/u: mindfulness-based cognitive therapy without follow-up sessions.

^d^STAI: State-Trait Anxiety Inventory.

^e^PAS: Panic and Agoraphobia Scale.

^f^LSAS: Liebowitz Social Anxiety Scale.

^g^EQ: Experiences Questionnaire.

^h^SF-36: Short-Form 36-Item Health Survey.

^i^SPANE: Scale of Positive and Negative Experience.

^j^RSES: Rosenberg Self-Esteem Scale.

^k^FFMQ: Five Facet Mindfulness Questionnaire.

^l^CDRISC: Connor Davidson Resilience Scale.

^m^SCS: Self-Compassion Scale.

^n^QIDS: 16-item Quick Inventory of Depressive Symptomatology.

^o^GAD7: Generalized Anxiety Disorder Assessment-7.

^p^PSS: Perceived Stress Scale.

^q^WHO-HPQ: World Health Organization Heath and Work Performance Questionnaire.

^r^SWLS: Satisfaction With Life Scale.

^s^FS: Flourishing Scale.

^t^MAIA: Multidimensional Assessment of Interoceptive Awareness.

^u^EQ-5D: EuroQoL-5 Dimensions.

^v^HAM-A: Hamilton Anxiety Scale.

### Sample Size

For a feasibility study that involves evaluating the standard deviation of continuous variables, a sample size of 24 to 50 cases is recommended [75,76]. Therefore, in this study, the maximum number of enrolled patients has been set to 50 (25 for each arm).

### Statistical Analysis

Statistical analyses and reporting of this trial will be conducted with primary analyses based on the intention-to-treat approach. The full analysis set will include all randomized subjects administered at least one procedure of the investigational treatment. For baseline variables, we will generate summary statistics with proportions and frequencies for categorical variables, and means and standard deviations for continuous data. Statistical data relevant to feasibility will be presented descriptively. For primary and secondary clinical outcome analyses, we will analyze mean changes from baseline with a restricted maximum likelihood-based repeated measures approach. The mixed model for repeated measures analyses will include the fixed and categorical effects of treatment, visit, and the treatment×visit interaction. We will employ Kenward-Roger approximation to estimate the degrees of freedom of the denominator. We will not conduct any adjustment for multiple testing of secondary outcomes because of the exploratory nature of the study. Imputation will not be performed for missing values because mixed models can deal with missing data by maximum likelihood. All comparisons are planned, and all P values are two-sided. A 5% significance level will be set for all statistical analyses. All statistical analyses will be conducted using Stata version 16 (Stata Corp).

### Subgroup Analysis

Considering the mixed participants in the study (those being offered face-to-face MBCT and the follow-up sessions online, and those being offered all sessions online), we intend to conduct a subgroup analysis sorted by participants receiving face-to-face MBCT and those receiving online MBCT.

### Adverse Events

When participants show serious adverse events, we will immediately contact the Ethics Review Committee at Keio University School of Medicine.

### Ethics

All procedures relevant to the study have been approved by the Ethics Review Committee of Keio University School of Medicine (reference number: 20190216). We have also ascertained that all procedures are in accordance with the ethical standards of the relevant national and institutional committees on human experimentation and with the Helsinki Declaration of 1975, as revised in 2008. The study is registered in the UMIN Clinical Trials Registry (UMIN000038626).

### Dissemination

We will present the results of the study at academic conferences, and the results are expected to be disseminated as articles in academic journals. The results of the study will comply with the CONSORT (Consolidated Standards of Reporting Trials) statement.

## Results

The study began to recruit participants in January 2020, and 43 participants have been enrolled up to January 2021. The intervention is ongoing and scheduled to be completed in February 2022. The participants in the first group (n=20) have been offered the 8-week MBCT intervention delivered in person. Owing to the COVID-19 pandemic, we were forced to switch to an online mode at the end of March 2020. Therefore, the participants in the first group were offered the face-to-face 8-week MBCT first, followed by online monthly follow-up sessions. For the second group (n=23), all interventions will be offered online. Data collection is expected to be concluded by May 2022.

## Discussion

The objective of this study is to investigate the long-term effectiveness of augmented MBCT with follow-up for anxiety disorders in comparison with MBCT without follow-up. Considering that the superiority of augmented MBCT posttreatment might not continue in the long term [30], developing a methodology to sustain its effectiveness in the long term is important. To the best of our knowledge, no studies to date have assessed the effectiveness of follow-up sessions after MBCT in comparison with MBCT alone. Therefore, this study is novel in terms of the design that compares augmented MBCT with and without follow-up sessions. Moreover, we expect our feasibility RCT to contribute to the development of well-designed definitive RCTs on the topic.

The limitations of our study are as follows. First, we expect differences in the participants included in the study in terms of the delivery of the intervention, as an impact of the COVID-19 pandemic (those offered face-to-face MBCT and online follow-up sessions, and those offered all sessions online). To account for the difference in the intervention delivery mode, we plan to conduct subgroup analysis. Second, we are not using a pharmacotherapy-alone arm. Thus, the study will not provide any implications regarding the clinical difference between augmented MBCT and pharmacotherapy alone. Nonetheless, considering that previous studies have already confirmed that augmented MBCT is superior to pharmacotherapy at posttreatment, we consider that our study design is acceptable from an ethical viewpoint. Despite the aforementioned limitations, we believe that this study will provide informative data for future clinical trials in this area.

## Abbreviations

CBT: cognitive behavioral therapy
CDRISC: Connor Davidson Resilience Scale
DSM-IV: Diagnostic and Statistical Manual of Mental Disorders, 4th edition
EQ: Experiences Questionnaire
EQ-5D: EuroQoL-5 Dimensions
FFMQ: Five Facet Mindfulness Questionnaire
FS: Flourishing Scale
GAD7: Generalized Anxiety Disorder Assessment-7
HAM-A: Hamilton Anxiety Scale
LSAS: Liebowitz Social Anxiety Scale
MAIA: Multidimensional Assessment of Interoceptive Awareness
MBCT: mindfulness-based cognitive therapy
MBSR: mindfulness-based stress reduction
PAS: Panic and Agoraphobia Scale
PSS: Perceived Stress Scale
QIDS: 16-item Quick Inventory of Depressive Symptomatology
RCT: randomized controlled trial
RSES: Rosenberg Self-Esteem Scale
SCID: Structured Clinical Interview for Diagnostic and Statistical Manual of Mental Disorders, 4th edition disorders
SCS: Self-Compassion Scale
SF-36: Short-Form 36-Item Health Survey
SPANE: Scale of Positive and Negative Experience
STAI: State-Trait Anxiety Inventory
SWLS: Satisfaction With Life Scale
WHO-HPQ: World Health Organization Heath and Work Performance Questionnaire

## References

[1] Stein DJ, Scott KM, de Jonge P, Kessler RC (2017). Epidemiology of anxiety disorders: from surveys to nosology and back. Dialogues Clin Neurosci.

[2] Baxter AJ, Vos T, Scott KM, Norman RE, Flaxman AD, Blore J, Whiteford HA (2014). The regional distribution of anxiety disorders: implications for the Global Burden of Disease Study, 2010. Int J Methods Psychiatr Res.

[3] Baxter AJ, Scott KM, Vos T, Whiteford HA (2013). Global prevalence of anxiety disorders: a systematic review and meta-regression. Psychol Med.

[4] Demyttenaere K, Bruffaerts R, Posada-Villa J, Gasquet I, Kovess V, Lepine JP, Angermeyer MC, Bernert S, de Girolamo G, Morosini P, Polidori G, Kikkawa T, Kawakami N, Ono Y, Takeshima T, Uda H, Karam EG, Fayyad JA, Karam AN, Mneimneh ZN, Medina-Mora ME, Borges G, Lara C, de Graaf R, Ormel J, Gureje O, Shen Y, Huang Y, Zhang M, Alonso J, Haro JM, Vilagut G, Bromet EJ, Gluzman S, Webb C, Kessler RC, Merikangas KR, Anthony JC, Von Korff MR, Wang PS, Brugha TS, Aguilar-Gaxiola S, Lee S, Heeringa S, Pennell BE, Zaslavsky AM, Ustun TB, Chatterji S, WHO World Mental Health Survey Consortium (2004). Prevalence, severity, and unmet need for treatment of mental disorders in the World Health Organization World Mental Health Surveys. JAMA.

[5] Kawakami N, Takeshima T, Ono Y, Uda H, Hata Y, Nakane Y, Nakane H, Iwata N, Furukawa TA, Kikkawa T (2005). Twelve-month prevalence, severity, and treatment of common mental disorders in communities in Japan: preliminary finding from the World Mental Health Japan Survey 2002-2003. Psychiatry Clin Neurosci.

[6] Kessler RC, Chiu WT, Demler O, Merikangas KR, Walters EE (2005). Prevalence, severity, and comorbidity of 12-month DSM-IV disorders in the National Comorbidity Survey Replication. Arch Gen Psychiatry.

[7] Casey B, Lee FS (2015). Optimizing treatments for anxiety by age and genetics. Ann N Y Acad Sci.

[8] Bruce SE, Yonkers KA, Otto MW, Eisen JL, Weisberg RB, Pagano M, Shea MT, Keller MB (2005). Influence of psychiatric comorbidity on recovery and recurrence in generalized anxiety disorder, social phobia, and panic disorder: a 12-year prospective study. Am J Psychiatry.

[9] Bandelow B, Michaelis S (2015). Epidemiology of anxiety disorders in the 21st century. Dialogues Clin Neurosci.

[10] Keller MB (2006). Social anxiety disorder clinical course and outcome: review of Harvard/Brown Anxiety Research Project (HARP) findings. J Clin Psychiatry.

[11] World Health Organization (2017). Depression and other common mental disorders: global health estimates.

[12] Greenberg PE, Sisitsky T, Kessler RC, Finkelstein SN, Berndt ER, Davidson JRT, Ballenger JC, Fyer AJ (1999). The economic burden of anxiety disorders in the 1990s. J Clin Psychiatry.

[13] McCrone P, Dhanasiri S, Patel A, Knapp M, Lawton-Smith S (2008). Paying the Price: The cost of mental health care in England to 2026.

[14] Sado M, Takechi S, Inagaki A, Fujisawa D, Koreki A, Mimura M, Yoshimura K (2013). Cost of anxiety disorders in Japan in 2008: a prevalence-based approach. BMC Psychiatry.

[15] (2005). Obsessive-compulsive disorder and body dysmorphic disorder: treatment. National Institute for Health and Care Excellence.

[16] (2019). Generalised anxiety disorder and panic disorder in adults: management. National Institute for Health and Care Excellence.

[17] (2013). Social anxiety disorder: recognition, assessment and treatment. National Institute for Health and Care Excellence.

[18] Stein MB, Roy-Byrne PP, Craske MG, Campbell-Sills L, Lang AJ, Golinelli D, Rose RD, Bystritsky A, Sullivan G, Sherbourne CD (2011). Quality of and patient satisfaction with primary health care for anxiety disorders. J Clin Psychiatry.

[19] Roy-Byrne P (2015). Treatment-refractory anxiety; definition, risk factors, and treatment challenges. Dialogues Clin Neurosci.

[20] Segal ZV, Williams JM, Teasdale JD (2012). Mindfulness-Based Cognitive Therapy for Depression: A New Approach to Preventing Relapse.

[21] Kabat-Zinn J (1990). Full Catastrophe Living: Using the Wisdom of Your Body and Mind to Face Stress, Pain, and Illness.

[22] Kocovski NL, Fleming JE, Hawley LL, Huta V, Antony MM (2013). Mindfulness and acceptance-based group therapy versus traditional cognitive behavioral group therapy for social anxiety disorder: a randomized controlled trial. Behav Res Ther.

[23] Donald J, Abbott MJ, Smith E (2014). Comparison of attention training and cognitive therapy in the treatment of social phobia: a preliminary investigation. Behav Cogn Psychother.

[24] Sundquist J, Lilja Å, Palmér K, Memon AA, Wang X, Johansson LM, Sundquist K (2015). Mindfulness group therapy in primary care patients with depression, anxiety and stress and adjustment disorders: randomised controlled trial. Br J Psychiatry.

[25] Goldin PR, Morrison A, Jazaieri H, Brozovich F, Heimberg R, Gross JJ (2016). Group CBT versus MBSR for social anxiety disorder: A randomized controlled trial. J Consult Clin Psychol.

[26] Koszycki D, Thake J, Mavounza C, Daoust J, Taljaard M, Bradwejn J (2016). Preliminary Investigation of a Mindfulness-Based Intervention for Social Anxiety Disorder That Integrates Compassion Meditation and Mindful Exposure. J Altern Complement Med.

[27] Wong SYS, Yip BHK, Mak WWS, Mercer S, Cheung EYL, Ling CYM, Lui WWS, Tang WK, Lo HHM, Wu JCY, Lee TMC, Gao T, Griffiths SM, Chan PHS, Ma HSW (2016). Mindfulness-based cognitive therapy v. group psychoeducation for people with generalised anxiety disorder: randomised controlled trial. Br J Psychiatry.

[28] Jazaieri H, Goldin PR, Werner K, Ziv M, Gross JJ (2012). A randomized trial of MBSR versus aerobic exercise for social anxiety disorder. J Clin Psychol.

[29] Ninomiya A, Sado M, Park S, Fujisawa D, Kosugi T, Nakagawa A, Shirahase J, Mimura M (2020). Effectiveness of mindfulness-based cognitive therapy in patients with anxiety disorders in secondary-care settings: A randomized controlled trial. Psychiatry Clin Neurosci.

[30] Eisendrath SJ, Gillung E, Delucchi KL, Segal ZV, Nelson JC, McInnes LA, Mathalon DH, Feldman MD (2016). A Randomized Controlled Trial of Mindfulness-Based Cognitive Therapy for Treatment-Resistant Depression. Psychother Psychosom.

[31] Segal ZV, Anderson AK, Gulamani T, Dinh Williams L, Desormeau P, Ferguson A, Walsh K, Farb NAS (2019). Practice of therapy acquired regulatory skills and depressive relapse/recurrence prophylaxis following cognitive therapy or mindfulness based cognitive therapy. J Consult Clin Psychol.

[32] Ribeiro L, Atchley RM, Oken BS (2018). Adherence to Practice of Mindfulness in Novice Meditators: Practices Chosen, Amount of Time Practiced, and Long-Term Effects Following a Mindfulness-Based Intervention. Mindfulness (N Y).

[33] First MB, Spitzer RL, Gibbon M, Williams JBW (1996). Structured Clinical Interview for DSM-IV Axis I Disorders (SCID-I).

[34] Spielberger CD (1983). Manual for the State-Trait Anxiety Inventory.

[35] Bandelow B (1995). Assessing the efficacy of treatments for panic disorder and agoraphobia. II. The Panic and Agoraphobia Scale. Int Clin Psychopharmacol.

[36] Rytwinski NK, Fresco DM, Heimberg RG, Coles ME, Liebowitz MR, Cissell S, Stein MB, Hofmann SG (2009). Screening for social anxiety disorder with the self-report version of the Liebowitz Social Anxiety Scale. Depress Anxiety.

[37] Fresco DM, Moore MT, van Dulmen MH, Segal ZV, Ma SH, Teasdale JD, Williams JMG (2007). Initial psychometric properties of the experiences questionnaire: validation of a self-report measure of decentering. Behav Ther.

[38] Fresco DM, Segal ZV, Buis T, Kennedy S (2007). Relationship of posttreatment decentering and cognitive reactivity to relapse in major depression. J Consult Clin Psychol.

[39] Brazier JE, Harper R, Jones NM, O'Cathain A, Thomas KJ, Usherwood T, Westlake L (1992). Validating the SF-36 health survey questionnaire: new outcome measure for primary care. BMJ.

[40] Diener E, Wirtz D, Tov W, Kim-Prieto C, Choi D, Oishi S, Biswas-Diener R (2009). New Well-being Measures: Short Scales to Assess Flourishing and Positive and Negative Feelings. Soc Indic Res.

[41] Rosenberg M (1965). Society and the adolescent self-image.

[42] Baer RA, Smith GT, Hopkins J, Krietemeyer J, Toney L (2006). Using self-report assessment methods to explore facets of mindfulness. Assessment.

[43] Connor KM, Davidson JR (2003). Development of a new resilience scale: the Connor-Davidson Resilience Scale (CD-RISC). Depress Anxiety.

[44] Neff KD (2003). The Development and Validation of a Scale to Measure Self-Compassion. Self and Identity.

[45] Rush A, Trivedi MH, Ibrahim HM, Carmody TJ, Arnow B, Klein DN, Markowitz JC, Ninan PT, Kornstein S, Manber R, Thase ME, Kocsis JH, Keller MB (2003). The 16-Item Quick Inventory of Depressive Symptomatology (QIDS), clinician rating (QIDS-C), and self-report (QIDS-SR): a psychometric evaluation in patients with chronic major depression. Biol Psychiatry.

[46] Spitzer RL, Kroenke K, Williams JBW, Löwe B (2006). A brief measure for assessing generalized anxiety disorder: the GAD-7. Arch Intern Med.

[47] Cohen S, Kamarck T, Mermelstein R (1983). A global measure of perceived stress. J Health Soc Behav.

[48] Kessler RC, Barber C, Beck A, Berglund P, Cleary PD, McKenas D, Pronk N, Simon G, Stang P, Ustun TB, Wang P (2003). The World Health Organization Health and Work Performance Questionnaire (HPQ). J Occup Environ Med.

[49] Diener E, Emmons RA, Larsen RJ, Griffin S (1985). The Satisfaction With Life Scale. J Pers Assess.

[50] Mehling WE, Price C, Daubenmier JJ, Acree M, Bartmess E, Stewart A (2012). The Multidimensional Assessment of Interoceptive Awareness (MAIA). PLoS One.

[51] Bornemann B, Herbert BM, Mehling WE, Singer T (2014). Differential changes in self-reported aspects of interoceptive awareness through 3 months of contemplative training. Front Psychol.

[52] Brooks R (1996). EuroQol: the current state of play. Health Policy.

[53] Hamilton M (1959). The assessment of anxiety states by rating. Br J Med Psychol.

[54] Shear MK, Vander Bilt J, Rucci P, Endicott J, Lydiard B, Otto MW, Pollack MH, Chandler L, Williams J, Ali A, Frank DM (2001). Reliability and validity of a structured interview guide for the Hamilton Anxiety Rating Scale (SIGH-A). Depress Anxiety.

[55] Koreki A, Garfkinel SN, Mula M, Agrawal N, Cope S, Eilon T, Gould Van Praag C, Critchley HD, Edwards M, Yogarajah M (2020). Trait and state interoceptive abnormalities are associated with dissociation and seizure frequency in patients with functional seizures. Epilepsia.

[56] Koreki A, Funayama M, Terasawa Y, Onaya M, Mimura M (2021). Aberrant Interoceptive Accuracy in Patients With Schizophrenia Performing a Heartbeat Counting Task. Schizophrenia Bulletin Open.

[57] Shimizu H, Imae K (1981). Development of the Japanese version of State-Trait Anxiety Inventory. Jpn. J. Educ. Psychol.

[58] Kaiya H, Ishii H, Masaki M, Komatsu C, Noguchi K, Sakai Y, Yoshida E, Kuribayashi K, Imaeda T (2017). Reliability and Validity of the Japanese Version of the Panic and Agoraphobia Scale (Patient). Anxiety Disord Res.

[59] Asakura S, Seishiro I, Fumi S, Yukiya S, Nobuki K, Takeshi I, Kenz D, Tsukasa K, Masumi I, Ryoji M (2002). Reliability and validity of the Japanese version of the Liebowitz Social Anxiety Scale. Seishin Igaku.

[60] Kurihara A, Hasegawa A, Nedate K (2010). Development of the Japanese Version of the Experiences Questionnaire and Examination of Its Reliability and Validity. The Japanese Journal of Personality.

[61] Fukuhara S, Ware JE, Kosinski M, Wada S, Gandek B (1998). Psychometric and Clinical Tests of Validity of the Japanese SF-36 Health Survey. Journal of Clinical Epidemiology.

[62] Sumi K (2013). Reliability and Validity of Japanese Versions of the Flourishing Scale and the Scale of Positive and Negative Experience. Soc Indic Res.

[63] Mimura C, Griffiths P (2007). A Japanese version of the Rosenberg Self-Esteem Scale: translation and equivalence assessment. J Psychosom Res.

[64] Sugiura Y, Sato A, Ito Y, Murakami H (2011). Development and Validation of the Japanese Version of the Five Facet Mindfulness Questionnaire. Mindfulness.

[65] Ito M, Nakajima S, Shirai A, Kim Y (2009). Cross-cultural validity of the Connor-Davidson Scale: data from Japanese population.

[66] Arimitsu K (2014). [Development and validation of the Japanese version of the Self-Compassion Scale]. Shinrigaku Kenkyu.

[67] Fujisawa D, Nakagawa A, Tajima M, Sado M, Kikuchi T, Iba M (2010). Cross-cultural adaptation of the quick inventory of depressive symptomatology-self report (QIDS-SR-J). Japanese Jornal of Stress Sciensce.

[68] Muramatsu K, Muramatsu Y, Miyaoka H, Fuse K, Yoshimine F (2010). Validation and utility of a Japanese version of the GAD-7. Japanese Journal of Psychosomatic Medicine.

[69] Mimura C, Griffiths P (2008). A Japanese version of the Perceived Stress Scale: cross-cultural translation and equivalence assessment. BMC Psychiatry.

[70] Suzuki T, Miyaki K, Sasaki Y, Song Y, Tsutsumi A, Kawakami N, Shimazu A, Takahashi M, Inoue A, Kurioka S, Shimbo T (2014). Optimal cutoff values of WHO-HPQ presenteeism scores by ROC analysis for preventing mental sickness absence in Japanese prospective cohort. PLoS One.

[71] Kadono T (1994). Development and validation of the Japanese version of the Satisfaction With Life Scale.

[72] Shoji M, Mehling WE, Hautzinger M, Herbert BM (2018). Investigating Multidimensional Interoceptive Awareness in a Japanese Population: Validation of the Japanese MAIA-J. Front Psychol.

[73] Tsuchiya A, Ikeda S, Ikegami N, Nishimura S, Sakai I, Fukuda T, Hamashima C, Hisashige A, Tamura M (2002). Estimating an EQ-5D population value set: the case of Japan. Health Econ.

[74] Yamamoto N, Aizawa R, Inagaki A, Inada T (2012). Inter-rater reliability of the Japanese version of the Structured Interview Guide for the Hamilton Anxiety rating scale (SIGH-A).

[75] Sim J, Lewis M (2012). The size of a pilot study for a clinical trial should be calculated in relation to considerations of precision and efficiency. J Clin Epidemiol.

[76] Julious SA (2005). Sample size of 12 per group rule of thumb for a pilot study. Pharmaceut. Statist.

